# Complete Maxillary Crossbite Correction with a Rapid Palatal Expansion in Mixed Dentition Followed by a Corrective Orthodontic Treatment

**DOI:** 10.1155/2016/8306397

**Published:** 2016-04-28

**Authors:** Orlando Motohiro Tanaka, Isabelle Adad Fornazari, Ariane Ximenes Graciano Parra, Bruno Borges de Castilhos, Ademir Franco

**Affiliations:** ^1^Graduate Dentistry Program in Orthodontics, Pontifícia Universidade Católica do Paraná, Rua Imaculada Conceição 1155, 80215-901 Curitiba, PR, Brazil; ^2^Postdoctoral Fellowship at The Center for Advanced Dental Education, Saint Louis University, St. Louis, MO, USA; ^3^Dentistry, Pontifícia Universidade Católica do Paraná, Curitiba, PR, Brazil; ^4^Orthodontics, Pontifícia Universidade Católica do Paraná, Curitiba, PR, Brazil; ^5^Department of Oral Health Sciences, Forensic Dentistry, KU Leuven and Dentistry, University Hospitals Leuven, Leuven, Belgium; ^6^Stomatology, Pontifícia Universidade Católica do Paraná, Curitiba, PR, Brazil

## Abstract

This case report presents the interceptive orthodontic treatment of a boy, aged 8 years 4 months with a Class I malocclusion with severe transverse maxillary deficiency and complete maxillary crossbite and correction using Haas expansion and fixed appliance. The treatment goals were to correct the posterior crossbite and anterior crossbite and restore the normality of the dentition and occlusion. In phase I, the patient was treated with a modified Haas-type palatal expander, which provided a clinically significant palatal expansion and increased the maxillary arch perimeter with favorable conditions for orthodontic treatment with fixed appliances in phase II. The optimization of E-space and the use of intermaxillary Class III elastics helped to maintain the mandibular incisors upright. A removable wraparound type appliance and a bonded lingual canine-to-canine retainer were used as retention. Although the literature has reported a high rate of relapse after palatal expansion, after 2 years 9 months of posttreatment follow-up, the occlusal result was stable and no skeletal reversals could be detected.

## 1. Introduction

The posterior crossbite is one of the most frequent malocclusions in orthodontics [1], and its possible etiologies include prolonged retention or loss at an early age of deciduous teeth, crowding, cleft palate, genetic factors, tooth-size arch-length discrepancies, abnormalities in tooth morphology, eruption sequence, thumb sucking habits, and mouth breathing during critical growth periods [2].

The rapid palatal expansion (RPE) is often used to expand the maxilla in patients with mixed dentition positively impacting the treatment of related deficiencies [3, 4]. Specifically, this technique may be used to correct transverse and sagittal crossbite, generating space in the dental arch and, consequently, solving cases of borderline crowding [5].

The RPE is extremely useful for the treatment of Class III patients and cases of real and relative maxillary deficiencies [6]. Occlusal acrylic splints are considered the most effective devices for RPE in young patients because they produce therapeutic effects that are not only limited to the correction of crossbite or the increase in arch width [5, 7].

Posterior crossbite and anterior crossbite do not have a spontaneous correction and should be treated with maxillary expansion as early as possible, after an accurate diagnosis with the patient in centric relation [8] and treatment planning accomplished with the patient compliance in using the appliance.

This paper aims to describe the great palatal expansion obtained after RPE, in mixed dentition, with the modified Haas palatal expander in a patient with complete maxillary crossbite and, after 2 years 9 months of posttreatment follow-up, the occlusal result was stable and no skeletal relapse could be detected.

## 2. Case Report

**Figure 1:**
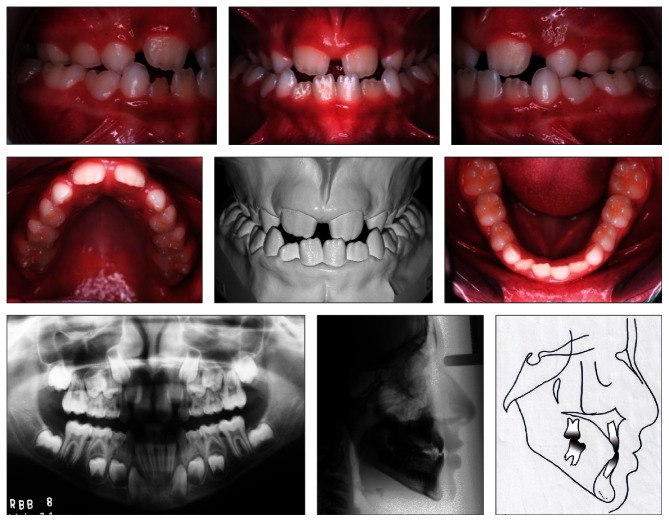
Pretreatment intraoral photographs, panoramic and cephalometric radiographs, and cephalometric tracing. Angle Class III malocclusion. Complete maxillary crossbite.

An 8.4-year-old male patient was referred by his general practitioner. On examination, the following factors were revealed: diastema between the central incisors and lack of space for lateral incisors eruption. Radiographically, the correct sequence of eruption, skeletal Class I malocclusion (ANB = 1°), with a Class III tendency (Ao-Bo = −8 mm) and angle Class III malocclusion, vertical growth trend, and upright maxillary and mandibular incisors were noted (Figure 1 and Table 1).

### 2.1. Treatment Objectives

The purpose of phase I was to correct the complete crossbite and indirectly increase the maxillary arch perimeter. The maintenance of the E-space in the mandibular arch with a lingual holding arch was recommended. The patient and parents were instructed that an undesirable growth pattern could occur based on the cephalometric measurements (Table 1) and also due to the genetic component (his father is skeletal Class III).

### 2.2. Treatment Alternatives

In phase I, the promptly correction of complete crossbite was advised, since the patient complied with the placement, use of the expanders, and especially the oral hygiene. The Haas, HYRAX, quad-helix, and bonded expander types were explained to the patient and his father.

### 2.3. Treatment Progress

**Figure 2:**
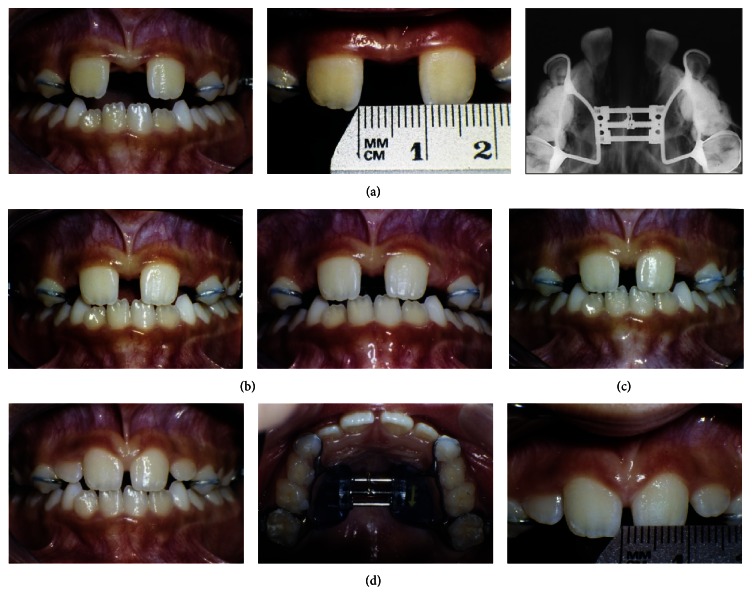
Midline diastema with clinical signs of the suture opening. Occlusal radiographs with real disjunction of the suture. (a) After three weeks and 52 screw activations. (b) 43 days and (c) and (d) 5 months and 6 months after retention. The diastema returned to the initial dimensions in the retention phase of the screw.

The choice was the Haas-type fixed palatal expander [3] with modification in the anterior region, due to the absence of premolars [6]. Both maxillary first permanent molars were banded and the expander was built with the 110M series (044-001, Summit Orthodontics). The expander was cemented in the permanent molars and bonded to the deciduous molars and canines. The orientation about care (food, hygiene, and activation) was explained after the palatal expander had been fixed (Figure 2).

**Figure 3:**
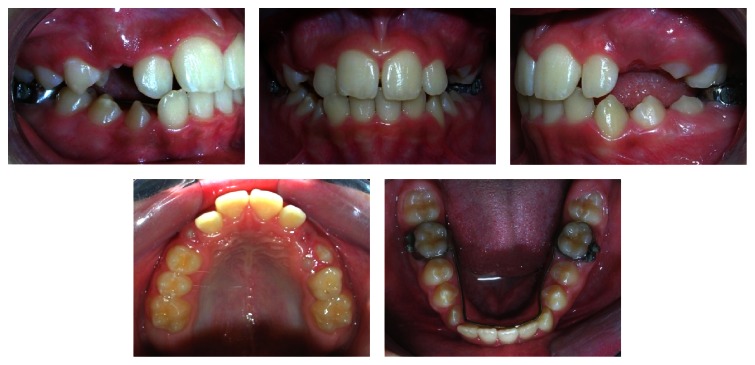
Lingual holding arch and the maintenance of the E-space.

Activation of expansion was performed with 2-quarter turns (0.5 mm) per day until the desired overexpansion was achieved, evaluated by the diastema opening and posterior transverse relationship on clinical observation. There was a very clinically significant opening of the diastema between the maxillary central incisors of 10.0 mm after three weeks and 52 screw activations. The occlusal radiograph shows the real disjunction of the sutures. The diastema returned to the initial dimensions at the retention phase (Figure 2). The expander was maintained as a retainer for a period of 6 months. The use of the remaining E-space enabled the alignment of the anterior lower teeth (Figure 3).

### 2.4. Treatment Results

In phase I, the RPE made the correction of the posterior crossbite and, concomitantly, the anterior crossbite possible due to the proclination of the maxillary incisors and uprighting of the lower incisors. There was also a clinically significant space gain in the maxillary dental arch allowing the alignment of the lateral incisors into the occlusion line, while the perimeter of the lower arch was maintained with the lingual holding arch (Figure 3).

**Figure 4:**
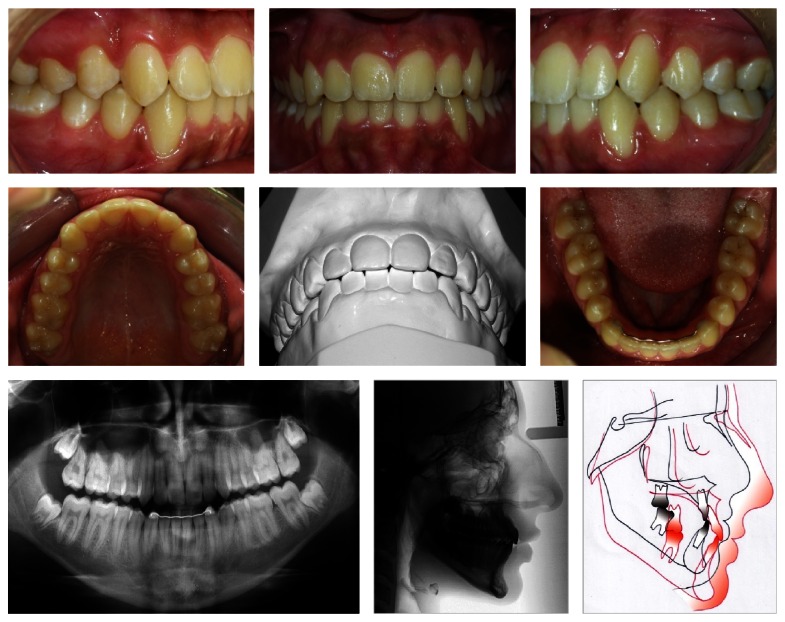
Posttreatment intraoral photographs. Good intercuspidation and adequate overjet and overbite. The skeletal and dental characteristics remained. Superimposition: pretreatment, black; posttreatment, red.

In phase II, at the age of 12 years 2 months, standard 0.022-in edgewise fixed appliance was recommended and the classic sequence of corrective treatment was applied with biomechanical control to minimize the proclination of the mandibular incisors. In the alignment and leveling stages, a Class III intermaxillary elastic on the left side and vertical elastics in the canine region on the right side were used to maintain and avoid canting the occlusal plane. The lingual arch was removed after the individual distalization of the premolars. Retraction and coordinated finishing arches were used. After removal of the fixed appliance, a removable wraparound type appliance in the maxillary arch and a wire segment were bonded lingually from canine to canine. The results of the alignment, leveling, and intercuspation are illustrated in Figure 4. Table 1 shows the maintenance of the skeletal pattern (ANB = 0°; Ao-Bo = −2 mm) and proclination of the maxillary incisor with the uprighting of the mandibular incisors. The patient had vertical growth favoring the stability of the occlusion.

**Figure 5:**
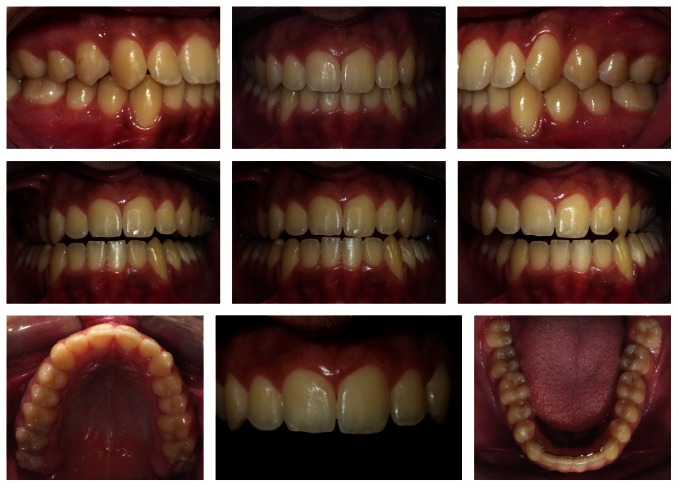
Two years nine months of posttreatment follow-up. Stability of the functional and occlusal results. Eruption of the third molars.

The functional and occlusal results were fully maintained at 2 years 9 months of posttreatment follow-up. All third molars were fully erupted (Figure 5).

## 3. Discussion

In the present case report, the patient had bilateral skeletal posterior crossbite, and a modified Haas-type expansion appliance was indicated. Early correction of posterior crossbite has been recommended in order to prevent inadequate skeletal transversal growth.

This clinical case did not present any difference between centric relation and maximum intercuspation confirming the differential diagnosis of complete maxillary crossbite, so the modified Haas expansion appliance was appointed [6]. Early age correction of posterior crossbite is recommended in order to prevent improper skeletal growth and to ensure the stability of the results.

The RPE performed in this clinical case was carried out in mixed dentition and reached maximum expansion with stability of the transverse dimension. The RPE promoted skeletal and dental positive effects enabling the correction of a transverse maxillary deficiency. Indirectly, it also corrected the anterior crossbite as noted by Haas, 1961 [9], on the projection of point “A,” with an increased SNA angle and an increased facial convexity angle, even if temporarily, causing the correction of the anterior crossbite. The choice of a great magnitude screw was another aspect of the clinically magnificent maxillary expansion. The maintenance of the Haas-type appliance as retention for 6 months can be considered as one of the stability factors as seen in this clinical case.

Although the correction of functional unilateral posterior crossbite can be achieved effectively at an early age with the quad-helix appliance [10] and treatment with the Haas-type expander, both are dependent on the cooperation of the patient and are in symbiosis with the diagnosis [11]. The success of the treatment is highly dependent on the degree of patient cooperation and motivation to accept unpleasant things, such as allowing adaptation, taking impressions, setting the expander, and properly brushing the teeth. They are also equally effective on the stability of the correction of posterior crossbite in increasing width and intermolar angulation [12].

In this clinical case, the activation was a 2-quarter turn a day. In theory, RPE applies a force on the posterior teeth, without giving enough time for the tooth movement to occur, so that the force is transferred to the sutures, resulting in a larger opening of the suture than teeth inclination [13]. The slow palatal expansion has advantages and disadvantages [14, 15]. Both, rapid and slow palatal expansion protocols cause buccal displacement of the first permanent maxillary molars, with more body displacement in the group with slow maxillary expansion, while more inclination in the group with RPE. Vertical and horizontal bone losses were noted in both groups; however, the slow expansion group had a great bone loss [14].

Despite the bonded expander and occlusal splint and the banded modified Haas type used in this clinical case, the response might be different. The vertical response of the posterior teeth appears to be greater in cases where the expansion was carried out without the occlusal stop [16]. This may also have favored the alignment and leveling of the lower teeth minimizing protruded maxillary incisors. The functional and esthetic results were fully achieved due to a combination of factors such as the significant arch-length perimeter, E-space optimization, vertical and sagittal proportional growth, and patient compliance.

## 4. Conclusion

The rapid palatal expansion using a modified Haas-type expander and the appropriate screw promotes positive skeletal (orthopedic) and dental (orthodontic) effects, affording, thus, the correction of a complete maxillary crossbite in mixed dentition. Favorable conditions have been provided for orthodontic treatment in permanent dentition with full braces obtaining function, facial and dental aesthetics, and the stability of the results.

## Figures and Tables

**Table 1:** Cephalometric measurements.

Measurements	Pretreatment 8.3	Progress 9.7	Posttreatment 17.2
SNA angle (°)	77	78	76
SNB angle (°)	76	78	76
ANB angle (°)	1	0	0
Ao-Bo (mm)	−8	−4	−2
Facial angle (°)	86	88	87
Convexity (°)	−3	−5	−7
FMA (°)	34	33	35
GoGn-SN (°)	43	40	41
*y*-axis (°)	61	59	61
1-NA (mm)	2	5	8
1-NA (°)	17	28	29
1-NB (mm)	4	5	5
1-NB (°)	22	14	18
Interincisal angle (°)	140	138	136
*Z*-angle (°)	71	71	75

## References

[1] Allen D., Rebellato J., Sheats R., Ceron A. M. (2003). Skeletal and dental contributions to posterior crossbites. *Angle Orthodontist*.

[2] Kutin G., Hawes R. R. (1969). Posterior cross-bites in the deciduous and mixed dentitions. *American Journal of Orthodontics*.

[3] Haas A. J. (1965). The treatment of maxillary deficiency by opening the midpalatal suture. *The Angle Orthodontist*.

[4] Wertz R. A. (1970). Skeletal and dental changes accompanying rapid midpalatal suture opening. *American Journal of Orthodontics*.

[5] McNamara J. A. (2002). Early intervention in the transverse dimension: is it worth the effort?. *American Journal of Orthodontics and Dentofacial Orthopedics*.

[6] Tanaka O., Orellana B., Ribeiro G. (2004). Singular aspects to operate rapid palatal expansion procedures. *Revista Dental Press de Ortodontia e Ortopedia Facial*.

[7] Vogel C. J. (2011). An interview with James A. McNamara Jr.. *Dental Press Journal of Orthodontics*.

[8] Celenza F. V. (1984). The theory and clinical management of centric positions: II. Centric relation and centric relation occlusion. *The International Journal of Periodontics & Restorative Dentistry*.

[9] Haas A. J. (1961). Rapid expansion of the maxillary dental arch and nasal cavity by opening the midpalatal suture. *The Angle Orthodontist*.

[10] Figueiredo M., Siqueira D., Bommarito S., Scanavini M. (2007). The early orthodontic treatment of posterior crossbites with attachment Quad-helix. *Revista Clínica de Ortodontia Dental Press*.

[11] Tanaka O., Maruo H., Camargo E. (2004). A intransponível grandeza do diagnóstico em Ortodontia. *Revista de Clínica e Pesquisa Odontológica*.

[12] Huynh T., Kennedy D. B., Joondeph D. R., Bollen A.-M. (2009). Treatment response and stability of slow maxillary expansion using Haas, hyrax, and quad-helix appliances: a retrospective study. *American Journal of Orthodontics and Dentofacial Orthopedics*.

[13] Haas A. J. (1980). Long-term posttreatment evaluation of rapid palatal expansion. *Angle Orthodontist*.

[14] Brunetto M., Da Silva Pereira Andriani J., Ribeiro G. L. U., Locks A., Correa M., Correa L. R. (2013). Three-dimensional assessment of buccal alveolar bone after rapid and slow maxillary expansion: a clinical trial study. *American Journal of Orthodontics and Dentofacial Orthopedics*.

[15] Lima Filho R. M. A., de Oliveira Ruellas A. C. (2008). Long-term maxillary changes in patients with skeletal Class II malocclusion treated with slow and rapid palatal expansion. *American Journal of Orthodontics and Dentofacial Orthopedics*.

[16] Miller C. L., Araújo E. A., Behrents R. G., Oliver D. R., Tanaka O. M. (2014). Mandibular arch dimensions following bonded and banded rapid maxillary expansion. *Journal of the World Federation of Orthodontists*.

